# Interoperability in the GENESIS 3.0 Software Federation: the NEURON Simulator as an Example

**DOI:** 10.1186/1471-2202-14-S1-P33

**Published:** 2013-07-08

**Authors:** Hugo Cornelis, Dimitris Bampasakis, Volker Steuber, James M Bower

**Affiliations:** 1 University of Texas Health Science Center, San Antonio, Texas, 78245, USA; 2 Barshop Institute for Longevity and Aging Studies, San Antonio, Texas, 78245, USA; 3 Science and Technology Research Institute, University of Hertfordshire, Hatfield, Hertfordshire, AL10 9AB, UK

## 

The idea of "interoperability" of neuroscience modeling software was instigated by the problems associated with incomplete model specifications in published papers and incremental model extensions through research projects across laboratories. Cannon et al. [1] defined interoperability as "all mechanisms that allow two or more simulators to use the same model description or to collaborate by evaluating different parts of a large neural model". As an example, the adoption of common declarative model definition languages such as *SBML*, *NeuroML*, and *NineML*, allows to simulate the same model on different simulator environments. Run-time interoperability allows different simulators to compute different aspects of the same model at run-time either by direct coupling via simulator script languages, indirect coupling via interpreted languages, or coupling via object oriented frameworks (see [2] for details).

The Computational Biology Initiative (CBI) federated software architecture is a software architecture that transparently supports both interoperability and "extensibility" for model building, simulation, and result analysis. It is a modular meta-framework for software development that integrates all the functions necessary for a fully functioning simulator. The modular nature of the CBI Architecture provides several advantages for multiple independent contributions to software development including: (1) Reduction in complexity of individual simulator components when compared to the complexity of a complete simulator, (2) Easy removal or replacement of unnecessary or obsoleted components, and (3) Clear delineation of the development scope of new components.

The CBI Architecture is designed to support alternative paradigms of interoperability and extensibility through the provision of logical relationships between its modules. The definition of a common information exchange reference model allows software modules to automatically interpret the information exchanged meaningfully and accurately in order to produce useful results as defined by end users such that any appropriately configured software component or application can be incorporated into the simulator.

GENESIS 3.0 (G-3) is a major reconfiguration and update of the GENESIS simulation system. G-3 is the first neural simulator to comply with the modular design of the CBI Architecture. It embodies many software components, each of which has been developed in full isolation. These include the **Model Container **that efficiently stores a representation of a model in computer memory and has simple bindings to the NeuroML and NineML declarative modeling languages, and **Heccer**: A fast compartmental solver based on the GENESIS *hsolve *object that can be instantiated from C, Python, Perl or other scripting languages. The **NS-SLI **is the G-3 component that provides backward compatibility for the GENESIS-2 SLI.

In this presentation we report on our recent efforts to reconfigure the NEURON simulator as an independent CBI compliant software component and to integrate its scripting interfaces with other CBI compliant software components of the GENESIS 3.0 neural simulation framework. The integration of selected aspects of the NEURON simulator into G-3 will allow to seamlessly integrate HOC model components with G-2 or G-3 model components. Employed in this way, the modular paradigm of the CBI Architecture supports interoperability by facilitating the functional integration of otherwise independent applications.
